# Intersections of sexual orientation, gender identity, and race/ethnicity and odds of reporting depression and anxiety symptomology in the Household Pulse Survey

**DOI:** 10.1080/09638237.2025.2558508

**Published:** 2025-09-13

**Authors:** Cody Ingle, RaeAnn Anderson, Andrew Williams

**Affiliations:** a Public Health Program, Department of Population Health, School of Medicine and Health Sciences, University of North Dakota, Grand Forks, ND, USA; b Department of Psychology, College of Arts & Sciences, University of North Dakota, Grand Forks, ND, USA

**Keywords:** Mental health, sexual orientation, transgender, gender identity, intersectionality

## Abstract

**Purpose:**

We examined odds of anxiety and depression symptomology among sexual and gender minority (SGM) individuals compared to straight and cisgender individuals, stratified by race.

**Methods:**

Data represented 918,892 households in the Household Pulse Survey from July 2021-October 2022. The Patient Health Questionnaire-2 measured depression symptoms (Scores >3=depression symptoms). The Generalized Anxiety Disorder Scale-2 measured anxiety symptoms (Scores >3=anxiety symptoms). Sexual orientation was categorical: “Gay/Lesbian,” “Straight,” “Bisexual,” “Something Else,” or “Don’t know.” Gender identity had 3 levels: “Cisgender Male,” “Cisgender Female,” or “Transgender/other gender identity.” Logistic regression estimated odds ratios(OR) and 95% confidence intervals(CI) for depression and anxiety among sexual minority individuals compared to straight individuals and transgender individuals compared to cisgender males. Intersection of sexual orientation/gender identity was also examined. Models (adjusted for sociodemographic factors) were stratified by race/ethnicity.

**Results:**

Transgender individuals showed doubled odds of depression (OR:2.30 95%CI:1.98,2.67) and anxiety (OR:2.41 95%CI:2.23,2.61) versus cisgender individuals. Bisexual individuals had nearly tripled odds versus straight individuals. Transgender bisexual individuals showed highest odds versus cisgender males (depression OR:6.22 95%CI:5.06,7.64; anxiety OR:7.11 95%CI:6.13,8.24). Non-Hispanic White individuals typically showed highest symptomology.

**Conclusion:**

SGM individuals showed increased anxiety and depression symptomology, with unexpected racial disparities warranting further intersectionality research.

## Introduction

Sexual and Gender Minority (SGM) people have historically been a group disproportionately affected by adverse health outcomes, including mental health (Rodriguez-Seijas et al., 2019; Vargas et al., 2020). Initial evidence from the Census Bureau’s, Household Pulse Survey (HPS) suggests that SGM individuals experience depression and anxiety at about twice the rate when compared to non-SGM individuals (File & Marlay, 2022).

SGM individuals experienced higher levels of anxiety and depression during COVID (Kneale & Bécares, 2021; Nowaskie & Roesler, 2022). Among 310 SGM individuals, 72% experienced depressive symptomology, and transgender individuals were approximately 20% more likely to experience depressive symptomology compared to cisgender individuals (race was not accounted for) (Kneale & Bécares, 2021). Additionally, recent data suggest 75% of gender minority people and 65% of cisgender sexual minority people disclosed worsening mental health during the COVID pandemic (Nowaskie & Roesler, 2022). While both studies provide data regarding mental health among SGM populations during the COVID-19 pan-demic, the sample sizes were small, and the intersectionality of race was unaccounted for.

### Intersectionality: a missing link

The social theory of intersectionality is often omitted from SGM research. Intersectionality focuses on mutually constitutive forms of social oppression rather than on single axes of difference (Hopkins, 2019). In this research, intersectionality will view categories of race, gender, and sexual orientation as overlapping, not isolated. Intersectionality highlights the unique experiences associated with overlapping identities. For example, the experience of Indigenous SGM individuals is distinct from that of SGM individuals racialized as Black, even though both groups are SGM people of color.

When considering mental health outcomes among SGM individuals, the intersecting identities of race and SGM status are important to analyze. Among a sample of 946 high school and college students aged 14–20, those who identified as SGM and youth of color were more likely to experience discrimination due to their identity, which in turn was associated with higher levels of depression, lower well-being, and lower grade point averages (Price et al., 2019). A nationwide study of United States high school students examined the 5-year trend (2015–2019) of the prevalence of feelings of sadness and hopelessness among various intersecting identifies of race/ethnicity and sex assigned at birth (Krause et al., 2022). An increase in the prevalence of feelings of sadness and hopelessness was observed, and Hispanic females had higher rates of sadness and hopelessness than other participants. However, the analysis did not account for SGM status (Krause et al., 2022).

Studies will often examine sexual minority individuals only and exclude gender minority individuals, even though many gender minority individuals also identify as sexual minority individuals (Depa et al., 2022). Furthermore, there is a lack of literature concerning intersectionality within the SGM community. When stratifying for race and compared to white SGM individuals, Black individuals reported decreased rates of mental health disorders (Rodriguez-Seijas et al., 2019). Minority stress theory hypothesizes that disparities in SGM populations are produced by excess exposure to social factors and is often utilized in SGM research (Frost & Meyer, 2023). When informed by intersectionality, one would hypothesize that multiple minority identities add to stressors faced by individuals. There is a need to study the intersection of race and SGM status to better understand the mental health outcomes of those with multiple minority identities. While there is growing literature on intersectionality and mental health, much is lacking concerning specific SGM populations.

### Study purpose

This study will add to the literature by examining depression and anxiety symptomology in the SGM community during the COVID-19 pandemic among individuals aged 18+ using an intersectional lens to understand racial differences.

Our focus on adults is significant because a majority of currently available studies look at youth (aged 13–24 years). In the intersectionality analysis, we hypothesized that odds of anxiety and depression symptomology would be stronger among SGM individuals that identify with a racially minoritized group than those that identify as White, and that gender minority individuals would have higher rates as well.

## Methods

### Data

The HPS is designed as a 20-minute online survey that measures household experiences during the COVID-19 pandemic. The survey asked questions mainly regarding the socioeconomic status of house-holds. As of July 21, 2021, the HPS became the first population survey sponsored by the Census Bureau to ask about sexual orientation and gender identity. The HPS used the Census Bureau’s Master Address File (MAF) to assign unique phone numbers and email addresses to one housing unit (HU) and utilizing the MAF to limit the specific HUs for the Contact Frame.

### Sample

The first wave of the HPS began on April 23, 2021. This sample includes individuals beginning in Wave 3.2 (Week 34), which started on July 21, 2021, due to the inclusion of Sexual Orientation and Gender Identity, until Wave 50 (ending October 17, 2022). All 17 weeks (34–50) were pooled together. The initial sample size for this group is N = 1,064,813. Respondents with missing data on any one of the variables of sexual orientation, gender identity, depression, anxiety, recent job loss, income, and education level were excluded. A total of 145,921 respondents were excluded for a final analytical sample size of N = 918,892.

### Outcome variables

#### Anxiety:

The HPS utilizes the two-question version of the Generalized Anxiety Disorder scale (GAD-2) for anxiety symptoms. The GAD-2 is a validated scale and has been validated in both primary care settings as well as the SGM community (Sapra et al., 2020). Participants answered two questions: “Over the last 2 weeks, how often have you been bothered by feeling nervous, anxious, or on edge” and “over the last 2 weeks, how often have you been bothered by not being able to stop or control worrying?” Each response is scored: Not at all (1); Several days (2); More than half the days(3); and Nearly every day(4). Scores for both questions are summed, with a score of 3 or greater indicating symptoms of Generalized Anxiety Disorder (Adams et al., 2022). Anxiety was categorized into a dichotomous variable: “Symptoms of Anxiety” or “No Symptoms of Anxiety.”

#### Depression:

the HPS utilizes the two-question version of the Patient Health Questionnaire (PHQ-2). The PHQ-2 is a validated measure that screens for Major Depressive Disorder and has been validated across the general population (Arrieta et al., 2017; Arroll et al., 2010). Participants answered two questions: “Over the last 2 weeks, how often have you been bothered by having little interest or pleasure in doing things” and then “Over the last 2 weeks, how often have you been bothered by feeling down, depressed, or hopeless?” Each response is scored: Not at all (1); Several days (2); More than half the days (3); and Nearly every day (4). Scores for both questions are summed, with a total score of 3 or greater indicating symptoms of Major Depressive Disorder. Depression was divided into dichotomous variables –“Symptoms of Depression” or “No Symptoms of Depression”

### Race, gender identity and sexual orientation variables

#### Race/ethnicity:

Participants Self-reported Hispanic origin and racial identity. A race/ethnicity variable was created using the “Hispanic origin” variable and the “Race” variable to identify racial/ethnic groups: “Hispanic, Latino, or Spanish”; “Non-Hispanic, White”; “Non-Hispanic, Black”; “Non-Hispanic, Asian,”; and “Non-Hispanic, Any other race or race combination.”

#### Gender Identity:

Participants were asked a series of questions regarding their gender identity. First, they were asked “What sex were you assigned at birth, on your original birth certificate?” Respondents could answer “Male” or “Female.” Next, participants were asked, “Do you currently describe yourself as male, female, or transgender” with the following choices: Male; Female; Transgender; None of these; Question seen but category not selected; Missing/did not report. For the purpose of this study, transgender was identified as anyone who’s sex at birth did not match their chosen gender or who chose transgender/none of these as their answer. Gender was divided into three categories: Cisgender Male; Cisgender Female; Transgender or other gender identity. Cisgender Male was used as the reference category.

#### Sexual Orientation:

Participants were asked to choose what best described their sexual identity. Participants were asked “Which of the following best represents how you think of yourself?” Participants selected from the following options: Gay or lesbian; Straight, that is not gay or lesbian; Bisexual; Other sexual minority; Questioning; Question seen but category not selected; Missing/Did not report. Respondents were categorized into 5 groups for sexual orientation: Gay or Lesbian; Straight; Bisexual; Other sexual minority; Questioning. “Other sexual minority” and “Questioning” responses were included to ensure that individuals who did not identify as “straight” were included in the analysis. Straight was used as the reference category.

#### Intersectionality:

Intersectionality was included in the models through the creation of 14 variables representing the full intersection of sexual orientation and gender identity, stratified by race.

### Covariates

Age (continuous), income level (categorical; “Less than $25,000,” “$25,000-$34,999,”” $35,000-$49,999,” “$50,000-$74,999,” “$75,000-$99,999,” “$100,000-$1149,999,” “150,000-$199,999,” “$200,000 and above”), recent job loss (categorical; “Yes,” “No,” “Question seen but category not selected,” “Missing/Did not report”), education level (categorical; “Less than high school,” “Some high school,” “High school graduate or equivalent,” “Some college but degree not received or is in progress,” “Associate’s degree,” “Bachelor’s degree,” “Graduate degree”), and region (categorical; “Northeast,” “South,” “Midwest,” “West”) were covariates informed by previous literature (Adams et al., 2022; Cai et al., 2021). Legislation regarding anti-SGM bills has been reported to affect mental health outcomes, making the region an important variable to consider for effect modification concerning mental health outcomes (Cunningham et al., 2022; Horne et al., 2022).

### Statistical analyses

Frequency (and percent) of depression and anxiety symptoms were reported by gender identity, sexual orientation, race/ethnicity and by covariates.

A series of logistic regression models were fit to estimate odds ratios (OR) and 95% Confidence intervals (95% CI) for the relationship between SGM status and symptoms of depression or anxiety. “Cisgender male” was used as the reference category for gender, which is supported by literature showing cisgender males exhibit lower depression and anxiety symptomology compared to other groups and is a group commonly used in logistic regression. (Ferlatte et al., 2020; Kirakosian et al., 2023; Toomey et al., 2018). First, a crude model was fit to estimate the baseline association between SGM status and depression and anxiety symptomology. Second, an adjusted model with age, education level, recent job loss, and income level was fit. In Model 3, region was added to the model to test for effect modification by geography. Next, to examine the role of intersectionality in odds of anxiety and depression symptomology, Models 1–3 were stratified by race/ethnicity in accordance with the categories created from survey respondents self-reported race and ethnicity. The domain function was utilized in SAS to stratify by race. Lastly, we examined the full intersection of sexual orientation/gender identity, stratified by race/ethnicity. In the initial analysis of the full intersection of sexual orientation/gender identity and race/ethnicity, “Cisgender male, straight” was used as the reference. In sensitivity analyses “Cisgender female, straight” was the reference to better illuminate potential gender-based mental health disparities.

Due to the number of models and comparisons in this analysis, we applied Bonferroni correct tests to fully adjusted models. Bonferroni correction tests Type 1 error by adjusting alpha number by the number of comparisons, making the statistical significance threshold stricter than 0.05 (Armstrong, 2014).

SAS allows replicated survey weights to be used, allowing a single sample to simulate multiple samples (80 replicate weights). Survey commands in SAS were used to factor replicate weights when performing the regression models. All statistical analyses were conducted using SAS OnDemand for Academics.

### Ethics

This project was deemed exempt via the procedures of the authors’ home institution Institutional Review Board at the University of North Dakota (IRB# 0005521). The project utilized deidentified secondary data from the Census Bureau’s Household Pulse Survey public use files.

### Patient and public involvement

No members of the public were involved in this original research. All data was public data analyzed from the Household Pulse Survey, which the Census Bureau distributed to households across the United States.

Interest in this study has been informed through multiple professional presentations that have discussed the suicidality and mental health of SGM individuals. When talking about suicide prevention, the SGM community is often mentioned as one of the highest-risk populations, particularly in rural areas. These presentations further developed an interest in this study.

### Consent

This study was conducted using secondary data publicly available and deidentified U.S. Census data, thus obtaining consent from survey participants was not possible nor deemed necessary by our institution’s IRB.

## Results

Table 1 contains descriptive characteristics of the sample. Out of 918,892 households examined in the survey, 31,662 (3.45%) identified as gay or lesbian, 33,948 (3.69%) identified as bisexual, 15,682 (1.5%) identified as other sexual minority individuals, and 14,574 (1.4%) identified as questioning. From 918,892 households, approximately 9.85% identified as a sexual orientation other than straight. 12,262 (1.33%) identified as transgender or other gender identity. The largest racial/ethnic group in the sample was non-Hispanic, White with 699,534 (76.13%), followed by Hispanic, Latino or Spanish 78,326 (8.52%), Non-Hispanic, Black 64,012 (6.97%), Non-Hispanic, Asian 43,649 (4.75%) and Non-Hispanic, Any other race or race combination 33,371 (3.63%). 58.90% of respondents reported symptoms of anxiety and 50.85% reported symptoms of depression.

### Depression and anxiety by gender identity

Table 2 includes the results for anxiety and depression symptomology by gender identity. The odds of reporting depression symptomology for transgender individuals was 2.61 (95% CI: 2.45, 2.78) times significantly greater than cisgender males after adjusting for all other independent variables in the model. When stratified by racial/ethnic group, transgender individuals had about two-fold higher odds compared to cisgender males aside from Non-Hispanic, Black individuals which had lower odds than the other groups, with 1.43 significantly greater odds (95% CI 1.41, 1.45).

The odds of reporting anxiety symptomology for transgender individuals was 2.24 (95% CI: 1.93, 2.61) times significantly greater than cisgender males after adjusting for all other independent variables in the model. All racial/ethnic groups had about 2–3 times increased odds of reporting symptoms of anxiety aside from Non-Hispanic, Black and Non-Hispanic, Asian, which both had lower odds.

### Depression and anxiety by sexual orientation

Regression results for anxiety and depression symptomology by sexual orientation are included in Table 3. Overall, all sexual minority groups had higher odds of reporting depression and anxiety symptomology compared to straight individuals. Bisexual individuals had the highest odds of depression symptomology of all sexual minority groups (OR:2.61, 95% CI 2.45, 2.78). All racial groups were elevated, aside from Non-Hispanic, Asian individuals who marked “Questioning” as their sexual orientation (OR 0.90, 95% CI 0.72, 0.86). Non-Hispanic, White individuals who identified as “Other sexual minority” had the highest odds of depression compared to other racial/ethnic groups (OR 2.33, 95% CI 2.28, 2.37).

The odds of anxiety symptomology among bisexual individuals was 2.83 (95% CI: 2.55, 3.15) times greater than straight individuals after adjusting for covariates All other racial/ethnic groups had elevated odds of anxiety except for Non-Hispanic, Asian individuals who marked “Questioning” as their sexual orientation (OR 0.82, 95% CI 0.76, 0.87).

Table 4a includes regression results for the full intersection of gender identity, sexual identity, and racial identity, with “Cisgender male, straight” as the reference. Among all gender identities and racial groups, bisexual individuals had the highest odds of reporting anxiety or depression symptomology. Transgender, bisexual individuals had 7.11 (95%CI: 6.13, 8.24) significantly higher odds of reporting anxiety symptomology and 6.22 (95%CI: 5.06, 7.64) significantly higher odds of reporting depression symptomology compared to cisgender, straight males. Odds of reporting anxiety and depression symptomology were mixed when observing the intersection of race, many individuals who identified as Non-Hispanic, White had higher odds of reporting anxiety or depression.

Table 4b includes regression results for the sensitivity analysis for the full intersection of gender identity, sexual identity, and racial identity, with “Cisgender female, straight” as the reference. Results were similar to initial analyses in that the majority of SGM experienced higher odds of anxiety and depression symptomology. However, the corresponding odds of anxiety and depression symptomology were lower than what was observed in the initial models in Table 4a.

For Tables 2–4, Bonferroni correction was run on the fully adjusted models due to the high number of regression models. After correction, most odds stayed statistically significant in Tables 2 and 3. This was not the case for Tables 4 (the full intersection of sexual orientation, gender identity, and race), in which multiple groups were not statistically significant after correction.

## Discussion

The purpose of this study was to examine the association between race, SGM status, and self-reported symptoms of anxiety and depression. Data shows that identifying as SGM is associated with an increased rate of self-reported symptoms of anxiety and depression, which supports our main hypothesis SGM individuals would have higher odds of anxiety and depression symptomology compared to cisgender and heterosexual individuals. In the intersectionality analyses addressed our second hypothesis that odds of anxiety and depression symptomology would be stronger among SGM individuals that identify with a racially minoritized group than those that identify as White was not fully supported. Results of the intersectionality analyses were mixed, as SGM individuals consistently had higher odds of anxiety and depression symptomology, but racially minoritized populations did not consistently have the highest odds.

All sexual minority groups had elevated odds of reporting anxiety and depression symptomology. However, bisexual individuals had the highest odds of anxiety and depression. Bisexual individuals having the highest odds were not expected in the initial research. However, this finding coincides with other data that shows bisexual individuals often report higher odds of depression and anxiety, which coincides with minority stress theory (La Roi et al., 2019). Due to the lack of social identity, bisexual individuals can feel as though they do not belong to a group and face discrimination within the SGM community (Postmes et al., 2019).

Overall, all gender minority individuals reported approximately two-fold higher odds of symptoms of anxiety and depression compared to cisgender males. This aligns with other studies that show heightened odds of depression, anxiety, and suicidality in gender minority populations (Fox et al., 2020; Nowaskie & Roesler, 2022; Ream, 2019).

Regarding the intersectionality of race and SGM status, Non-Hispanic, Black individuals had lower odds of reporting anxiety or depression symptomology compared to Non-Hispanic, White individuals that identified as SGM. Literature suggests that multiple layers of minority status have been associated with increased rates of depression and anxiety symptomology (Adams et al., 2022; Casey et al., 2022). Adams et al. used HPS data to show that minority ethnic groups have multiple layers of stress, particularly minority stress, that contributes to their mental health. This study showed through stratification of race/ethnicity that intersecting identities of race/ethnicity and SGM status does have an effect on anxiety and depression symptomology. The data also showed that Non-Hispanic, White individuals had higher odds compared to some other racial groups (Non-Hispanic, Asian and No-Hispanic, Black in some cases). These results coincide with the literature that race is a complicated role in depression and anxiety symptomology.

When examining the full intersection of race, sexual identity, and gender identity, there are significantly higher odds among almost all SGM groups compared to cisgender, straight males. Some groups had wide confidence intervals, which indicates low sample size. The low statistical power of groups with small sample size, coupled with stricter significance threshold of the Bonferroni correction explains why many groups were not statistically significant after the Bonferroni correction. In sensitivity analysis, using “Cisgender female, straight” as the reference group resulted in similar observation that SGM had higher odds of depression and anxiety symptomology. Given the lack of statistical significance for many groups after Bonferroni correction, the observed elevated odds of anxiety and depression symptomology among SGM groups indicates the necessity of gathering more data on the intersection of race and SGM status.

Some studies have found that having a positive view of racial identity/group is a protective factor for negative mental health outcomes (Brance et al., 2023; Salerno et al., 2020). Brance et al. found that social identification, a positive view and integration of one’s race/ethnicity into their full identity, is a protective factor against common psychological disorders but with smaller effect sizes for anxiety and depression (though the protective factor still remains). One meta-analysis found that higher social identification is linked to lower levels of depression but states that the relationship is complex (Postmes et al., 2019).

The idea of social identification as a protective factor could also be extrapolated to SGM communities. This study found that Bisexual individuals have significantly higher odds compared to other sexual minority groups, which could potentially be explained by a lack of social identification. As discussed, bisexual individuals often do not feel identified with either community (straight or SGM) (La Roi et al., 2019).

### Limitations

Limitations of this study include limited data on sexual orientation and gender identity (i.e. gay and lesbian combined into one category), thus limiting how transgender individuals may be categorized (i.e. only including “transgender” as an option for transgender individuals regarding gender identity). Limited racial information was gathered in the HPS as well, so more information is needed in order to fully assess intersecting identities. For example, Indigenous people and multiracial people were labeled as the same category. However, survey results were self-reported, so individuals likely reported based on their identity.

### Strengths

Strengths of this study include the HPS being the first Census Bureau product to collect data on sexual orientation and gender identity. Demographic statistics concerning SGM populations coincide with population-level estimates of SGM numbers in the United States estimated by Gallup, thus suggesting the Pulse survey is a good representation of SGM in the US. Utilization of the PHQ-2 and GAD-2 as validated measures of symptoms of anxiety and depression strengthened the validity of measurement tools.

## Conclusion

This study showed the need for mental health interventions that specifically focus on intersecting identities, including strategies for positive social interaction and identification. All sexual minority individuals had elevated odds of reporting anxiety and depression symptomology, but Bisexual individuals had the highest odds of reporting depression symptomology among sexual minority groups. Non-Hispanic, Black individuals mostly reported lower odds among SGM, which was not initially expected. Results also show the need for SGM-specific training for mental health providers to help mitigate depression and anxiety symptomology. More research is necessary to fully understand the effect intersectionality has on mental health of SGM populations.

## Figures and Tables

**Table 1.** Descriptive statistics for symptoms of depression and anxiety from the Household Pulse Survey (*n* = 918,892).

Variable	Overall Unweighted N (unweighted %)	Depression		Anxiety	
		Symptoms of Depression Unweighted N (unweighted %)	No Symptoms of Depression Unweighted N (unweighted %)	Symptoms of Anxiety Unweighted N (unweighted %)	No Symptoms of Anxiety Unweighted N (unweighted %)

**Gender Identity**					
Cisgender Male	425,148 (40.48)	171,703 (45.32)	207,140 (54.68)	187,513 (49.46)	191,634 50.54
Cisgender Female	610,053 (58.09)	292,01 (54.16)	247,183 (45.84)	350,794 (65.00)	188,861 (35.00)
Transgender or other gender identity	15,021 (1.43)	8,863 (71.23)	3,580 (28.77)	9,461 (75.91)	3,003 (24.09)
**Sexual Orientation**					
Straight	940,739 (90.09)	406,419 (48.67)	428,610 (51.33)	474,984 (56.84)	360,719 (43.16)
Gay or Lesbian	34,901 (3.34)	19,823 (62.31)	11,993 (37.69)	22,180 (69.68)	9,653 (30.32)
Bisexual	38,296 (3.67)	26,332 (77.27)	7,746 (22.73)	28,915 (84.82)	5,176 (15.18)
Other Sexual Minority	15,682 (1.50)	10,305 (75.50)	3,344 (24.50)	11,111 (81.29)	2,558 (18.71)
Questioning	14,574 (1.40)	7,767 (66.80)	3,860 (33.20)	8,299 (71.22)	3,353 (28.78)
**Race/Ethnicity**					
Non-Hispanic, White	795,321 (74.69)	352,471 (49.49)	359,669 (50.51)	413,073 (57.95)	299,702 (42.05)
Hispanic, Latino, or Spanish	97,751 (9.18)	46,526 (57.66)	34,163 (42.34)	52,601 (65.11)	28,187 (34.89)
Non-Hispanic, Black	80,060 (7.52)	35,088 (53.37)	30,653 (46.63)	38,952 (59.22)	26,820 (40.78)
Any other race or race in combination	39,430 (3.70)	20,819 (61.24)	13,176 (38.76)	23,362 (68.65)	10,667 (31.35)
**Income**					
Less than $25,000	26,872 (3.06)	65,721 (69.95)	28,238 (30.05)	69,143 (73.54)	24,883 (26.46)
$25,000-$34,999	75,287 (8.57)	46,902 (62.59)	28,029 (37.41)	50,186 (66.94)	24,787 (33.06)
$35,000-$49,999	91,608 (10.42)	53,166 (58.25)	38,102 (41.75)	58,276 (63.82)	33,033 (36.18)
$50,000-$74,999	144,267 (16.42)	76,128 (52.94)	67,676 (47.06)	86,424 (60.07)	57,456 (39.93)
$75,000-$99,999	120,639 (13.73)	58,427 (48.58)	61,851 (51.42)	68,674 (57.07)	51,665 (42.93)
$100,000-$149,999	154,362 (17.56)	68,250 (44.34)	85,663 (55.66)	84,000 (54.54)	70,009 (45.46)
150,000-$199,999	6,332 (8.69)	30,835 (40.50)	45,299 (59.50)	39,524 (51.90)	36,629 (48.10)
$200,000 and above	94,996 (10.81)	34,434 (36.35)	60,292 (63.65)	45,993 (48.53)	48,788 (51.47)
**Education Level**					
Less than high school	7173 (0.67)	3,205 (60.06)	2,131 (39.94)	3,276 (61.23)	2,074 (38.77)
Some high school	14,805 (1.39)	6,839 (60.93)	4,385 (39.07)	7,126; 63.30	4,131 (36.70)
High school graduate or equivalent	125,022 (1.74)	55,746 (54.21)	47,078 (45.79)	59,531 (57.83)	43,406 (42.17)
Some college, but degree not received or is in progress	224,070 (21.04)	109,533 (56.78)	83,371 (43.22)	119,921 (62.13)	73,096 (37.87)
Associate’s degree	111,070 (10.43)	52,475 (54.17)	44,395 (45.83)	58,813 (60.66)	38,150 (39.34)
Bachelor’s degree	305,631 (28.70)	135,022 (49.20)	139,400 (50.80)	160,421 (58.41)	114,234 (41.59)
Graduate degree	277,042 (26.02)	112,734 (44.45)	140.859 (55.55)	142,016 (55.92)	111,823 (44.05)
**Recent Household Job Loss (last 4 weeks)**					
Yes	111,448 (10.86)	72,919 (74.48)	24,982 (49.84)	79,400 (81.01)	18,617 (18.99)
No	926,208 (88.57)	401,869 (47.97)	435,876 (52.03)	470,852 (56.16)	367,602 (43.84)

**Table 2.** Odds of depression and anxiety symptomology by gender identity.

Depression						
Gender Identity	Overall OR (95% CI)^d^	Hispanic OR (95% CI)	Non-Hispanic White OR (95% CI)	Non-Hispanic Black OR (95% CI)	Non-Hispanic Asian OR (95% CI)	Any other race or race combination OR (95% CI)

Model 1^a^						
Cisgender Male	Ref.^e^	Ref.	Ref.	Ref.	Ref.	Ref.
Cisgender Female	1.30 (1.27, 1.33)	1.28 (1.26, 1.29)	1.29 (1.24, 1.33)	1.34 (1.30, 1.37)	1.32 (1.30, 1.35)	1.32 (1.25, 1.39)
Transgender or other gender identity	2.96 (2.58, 3.41)	2.21 (2.00, 2.48)	3.67 (3.07, 4.39)	1.82 (1.76, 1.88)	2.19 (1.66, 2.90)	2.30 (1.71, 3.10)
Model 2^b^						
Cisgender Male	Ref.	Ref.	Ref.	Ref.	Ref.	Ref.
Cisgender Female	1.24 (1.20, 1.29)	1.22 (1.16, 1.28)	1.25 (1.22, 1.28)	1.21 (1.15, 1.27)	1.31 (1.26, 1.35)	1.25 (1.17, 1.34)
Transgender or other gender identity	2.08 (1.62, 2.67)	2.20 (1.56, 3.09)	2.13 (1.84, 2.47)	1.43 (1.41, 1.45)	1.89 (1.32, 2.71)	2.15 (1.13, 4.09)
Model 3^c^						
Cisgender Male	Ref.	Ref.	Ref.	Ref.	Ref.	Ref.
Cisgender Female	1.24 (1.20, 1.29)*	1.22 (1.16, 1.28)*	1.25 (1.22, 1.28)*	1.21 (1.15, 1.27)*	1.30 (1.26, 1.34)*	1.25 (1.17, 1.35)*
Transgender or other gender identity	2.08 (1.62, 2.67)*	2.20 (1.56, 3.08)*	2.13 (1.84, 2.47)*	1.43 (1.41, 1.45)*	1.91 (1.33, 2.74)*	2.15 (1.14, 4.07)
Anxiety						
Gender Identity						
Model 1^a^						
Cisgender Male	Ref.	Ref.	Ref.	Ref.	Ref.	Ref.
Cisgender Female	1.70 (1.63, 1.76)	1.58 (1.56, 1.61)	1.71 (1.63, 1.81)	1.70 (1.63, 1.78)	1.73 (1.62, 1.84)	1.77 (1.77, 1.77)
Transgender or other gender identity	3.11 (2.99, 3.23)	2.31 (2.07, 2.57)	3.82 (3.63, 4.02)	2.04 (1.79, 2.33)	1.99 (1.42, 2.80)	2.89 (2.85, 2.92)
Model 2^b^						
Cisgender Male	Ref.	Ref.	Ref.	Ref.	Ref.	Ref.
Cisgender Female	1.70 (1.65, 1.76)	1.55 (1.51, 1.59)	1.76 (1.72, 1.80)	1.58 (1.51, 1.66)	1.71 (1.61, 1.83)	1.76 (1.70, 1.82)
Transgender or other gender identity	2.24 (1.93, 2.61)	2.35 (1.73, 3.19)	2.14 (2.14, 2.15)	1.87 (1.85, 1.88)	1.83 (1.25, 2.67)	2.89 (2.00, 4.17)
Model 3^c^						
Cisgender Male	Ref.	Ref.	Ref.	Ref.	Ref.	Ref.
Cisgender Female	1.70 (1.65, 1.76)*	1.55 (1.51, 1.59)*	1.80 (1.72, 1.80)*	1.58 (1.51, 1.66)*	1.71 (1.61, 1.82)*	1.76 (1.70, 1.83)*
Transgender or other gender identity	2.24 (1.92, 2.61)*	2.35 (1.72, 3.21)*	2.14 (2.14, 2.15)*	1.87 (1.85, 1.88)*	1.84 (1.25, 2.70)	2.89 (2.01, 4.15)*

a Crude Model.

b Adjusted for age, income, education level, and recent job loss.

c Adjusted for age, income, education level, recent job loss, and region.

d odds ratio (95% confidence interval).

e Reference category.

* OR statistically significant after Bonferroni correction.

**Table 3.** Odds of depression and anxiety symptomology by sexual orientation.

Depression						
Sexual Orientation	Overall OR (95% CI)	Hispanic OR (95% CI)	Non-Hispanic White OR (95% CI)	Non-Hispanic Black OR (95% CI)	Non-Hispanic Asian OR (95% CI)	Any other race or race combination OR (95% CI)

Model 1^a^						
Straight	Ref.	Ref.	Ref.	Ref.	Ref.	Ref.
Gay or Lesbian	2.01 (1.86, 2.18)	1.65 (1.49, 1.82)	2.15 (1.94, 2.38)	1.77 (1.66, 1.88)	2.20 (2.01, 2.40)	1.60 (1.50, 1.72)
Bisexual	4.04 (3.80, 4.29)	3.52 (3.19, 3.88)	4.36 (3.93, 4.84)	2.90 (2.84, 2.96)	2.98 (2.97, 2.99)	3.96 (3.19, 4.93)
Other sexual minority	2.89 (2.88, 2.90)	1.54 (1.47, 1.62)	3.86 (3.73, 4.00)	2.59 (1.96, 3.43)	2.52 (2.40, 2.64)	2.24 (1.75, 2.86)
Questioning	1.79 (1.63, 1.97)	1.39 (1.28, 1.51)	2.41 (2.28, 2.55)	1.26 (1.22, 1.31)	1.03 (1.03, 1.04)	1.40 (1.06, 1.85)
Model 2^b^						
Straight	Ref.	Ref.	Ref.	Ref.	Ref.	Ref.
Gay or Lesbian	1.71 (1.64, 1.77)	1.52 (1.52, 1.53)	1.76 (1.68, 1.84)	1.62 (1.51, 1.74)	1.92 (1.62, 2.27)	1.37 (1.30, 1.45)
Bisexual	2.61 (2.45, 2.78)	2.71 (2.37, 3.09)	2.56 (2.36, 2.78)	2.14 (2.05, 2.24)	2.21 (2.02, 2.42)	2.89 (2.41, 3.45)
Other sexual minority	1.97 (1.89, 2.05)	1.37 (1.05, 1.81)	2.33 (2.28, 2.37)	2.12 (1.98, 2.27)	1.75 (1.73, 1.77)	1.90 (1.25, 2.90)
Questioning	1.32 (1.16, 1.51)	1.22 (1.07, 1.39)	1.70 (1.59, 1.82)	1.16 (0.96, 1.40)	0.90 (0.72, 0.86)	1.31 (1.25, 1.37)
Model 3^c^						
Straight	Ref.	Ref.	Ref.	Ref.	Ref.	Ref.
Gay or Lesbian	1.71 (1.64, 1.77)*	1.53 (1.52, 1.53)*	1.76 (1.68, 1.84)*	1.62 (1.50, 1.74)*	1.90 (1.61, 2.24)*	1.38 (1.30, 1.46)*
Bisexual	2.61 (2.45, 2.78)*	2.71 (2.37, 3.09)*	2.56 (2.36, 2.78)*	2.14 (2.05, 2.24)*	2.20 (2.01, 2.40)*	2.89 (2.42, 3.45)*
Other sexual minority	1.97 (1.89, 2.05)*	1.37 (1.05, 1.80)	2.33 (2.28, 2.37)*	2.11 (1.97, 2.27)*	1.76 (1.71, 1.81)*	1.90 (1.25, 2.90)
Questioning	1.32 (1.16, 1.51)	1.22 (1.08, 1.39)	1.70 (1.59, 1.82)*	1.16 (0.96, 1.40)	0.80 (0.74, 0.87)	1.31 (1.25, 1.37)*
Anxiety						
Sexual Orientation						
Model 1^a^						
Straight	Ref.	Ref.	Ref.	Ref.	Ref.	Ref.
Gay or Lesbian	2.10 (1.89, 2.35)	1.87 (1.77, 1.98)	2.22 (1.92, 2.57)	1.66 (1.57, 1.76)	2.20 (2.09, 2.31)	1.81 (1.69, 1.93)
Bisexual	4.69 (4.39, 5.00)	3.91 (3.63, 4.22)	5.31 (4.75, 5.94)	3.15 (2.95, 3.37)	2.74 (2.41, 3.10)	3.85 (3.34, 4.43)
Other sexual minority	2.85 (2.35, 3.46)	1.37 (1.10, 1.69)	4.10 (3.89, 4.33)	2.88 (2.60, 3.19)	2.24 (2.07, 2.41)	2.33 (2.08, 2.61)
Questioning	1.59 (1.42, 1.79)	1.13 (0.99, 1.28)	2.38 (2.14, 2.63)	1.17 (0.94, 1.45)	0.93 (0.90, 0.97)	1.17 (0.96, 1.44)
Model 2^b^						
Straight	Ref.	Ref.	Ref.	Ref.	Ref.	Ref.
Gay or Lesbian	1.67 (1.55, 1.80)	1.59 (1.59, 1.59)	1.67 (1.53, 1.83)	1.52 (1.41, 1.63)	1.91 (1.66, 2.20)	1.56 (1.55, 1.57)
Bisexual	2.83 (2.55, 3.15)	3.03 (2.70, 3.39)	2.78 (2.51, 3.07)	2.27 (2.15, 2.39)	2.16 (2.0, 2.34)	2.68 (2.17, 3.31)
Other sexual minority	1.94 (1.68, 2.23)	1.32 (1.28, 1.36)	2.31 (2.06, 2.59)	2.44 (2.02, 2.95)	1.61 (1.44, 1.81)	1.97 (1.75, 2.20)
Questioning	1.21 (1.08, 1.36)	1.17 (1.16, 1.19)	1.63 (1.52, 1.75)	1.08 (0.97, 1.19)	0.82 (0.76, 0.87)	1.17 (1.09, 1.26)
Model 3^c^						
Straight	Ref.	Ref.	Ref.	Ref.	Ref.	Ref.
Gay or Lesbian	1.67 (1.55, 1.80)*	1.59 (1.58, 1.59)*	1.67 (1.53, 1.83)*	1.51 (1.41, 1.63)*	1.90 (1.66, 2.18)*	1.56 (1.55, 1.58)*
Bisexual	2.83 (2.55, 3.15)*	3.03 (2.69, 3.40)*	2.79 (2.51, 3.08)*	2.27 (2.15, 2.39)*	2.15 (2.0, 2.32)*	2.68 (2.17, 3.31)*
Other sexual minority	1.94 (1.69 2.23)*	1.32 (1.29, 1.36)*	2.31 (2.07, 2.59)*	2.44 (2.02, 2.95)*	1.62 (1.46, 1.79)*	2.00 (1.76, 2.20)*
Questioning	1.21 (1.08, 1.36)*	1.11 (0.92, 1.35)	1.63 (1.52, 1.75)*	1.08 (0.97, 1.19)	0.93 (0.77, 0.87)*	1.17 (1.09, 1.12)

a Crude Model.

b Adjusted for age, income, education level, and recent job loss.

c Adjusted for age, income, education level, recent job loss, and region.

* OR statistically significant after Bonferroni correction.

**Table 4.** Odds of depression and anxiety symptomology by sexual orientation, gender identity, and race/ethnicity intersections.

a. Cisgender male, straight as reference.						
Anxiety						
Sexual Orientation, Gender Identity, and Race/ethnicity intersection	Overall OR (95% CI)	Hispanic OR (95% CI)	Non-Hispanic White OR (95% CI)	Non-Hispanic Black OR (95% CI)	Non-Hispanic Asian OR (95% CI)	Any other race or race combination OR (95% CI)

Fully Adjusted Model^a^						
Cisgender Male, Straight	Ref.	Ref.	Ref.	Ref.	Ref.	Ref.
Cisgender Male, Gay	1.95 (1.87, 2.04)*	1.88 (1.75, 2.02)	1.94 (1.81, 2.09)	1.86 (1.77, 1.94)*	1.85 (1.73, 1.98)	2.13 (1.96, 2.28)*
Cisgender Male, Bisexual	2.36 (2.00, 2.79)	3.12 (1.82, 5.33)	2.28 (2.12, 2.44)*	1.75 (1.49, 2.07)	1.47 (1.26, 1.72)	2.03 (1.38, 2.98)
Cisgender male, Other sexual minority	1.85 (1.82, 1.89)*	1.49 (1.42, 1.56)	2.16 (1.94, 2.40)	2.13 (1.27, 3.59)	1.49 (1.38, 1.61)	1.29 (0.73, 2.26)
Cisgender Male, Questioning	1.33 (1.02, 1.72)	1.34 (0.75, 2.38)	1.81 (1.78, 1.84)*	1.09 (1.09, 1.09)*	0.96 (0.76, 1.20)	0.88 (0.46, 1.69)
Cisgender Female, Straight	1.71 (1.70, 1.75)*	1.62 (1.58, 1.66)*	1.76 (1.73, 1.79)*	1.59 (1.55, 1.63)*	1.71 (1.64, 1.78)*	1.74 (1.69, 1.80)*
Cisgender Female, Lesbian	2.56 (2.35, 2.79)*	2.61 (1.77, 3.87)	2.64 (1.57, 2.71)*	2.00 (1.81, 2.13)	4.08 (3.55, 4.68)	1.84 (1.64, 2.07)
Cisgender Female, Bisexual	4.53 (4.34, 4.72)*	4.09 (3.40, 4.93)	4.58 (4.15, 5.05)*	3.76 (3.62, 3.92)*	4.28 (3.67, 4.97)	4.06 (3.92, 4.20)*
Cisgender Female, Other sexual minority	2.81 (2.36, 3.35)	1.39 (1.09, 1.77)	4.16 (4.04, 4.29)*	4.45 (3.24, 6.13)	2.05 (1.95, 2.15)*	3.98 (3.83, 4.14)*
Cisgender Female, Questioning	1.73 (1.44, 2.07)	1.11 (0.95, 1.30)	2.78 (2.24, 3.46)	2.00 (1.23, 3.24)	1.22 (1.00, 1.50)	1.49 (1.44, 1.53)*
Transgender/Other Gender Identity, Straight	1.29 (1.13, 1.47)	1.53 (1.14, 2.04)	1.20 (1.14, 1.25)	1.56 (1.46, 1.66)	1.37 (0.91, 2.04)	1.31 (1.04, 1.65)
Transgender/Other Gender Identity, Gay or Lesbian	4.32 (3.76, 4.95)	1.58 (1.03, 2.42)	6.24 (4.44, 8.76)	7.72 (5.76, 10.35)	18.70 (5.26, 66.51)	3.16 (2.48, 4.03)
Transgender/Other Gender Identity, Bisexual	7.11 (6.13, 8.24)*	6.83 (5.82, 8.02)*	7.00 (6.03, 8.12)*	4.61 (4.60, 4.63)*	3.43 (3.22, 3.64)*	9.31 (3.34, 26.00)
Transgender/Other Gender Identity, Other Sexual Minority	3.10 (2.08, 4.64)	2.78 (2.23, 3.47)	2.85 (1.46, 5.55)	3.24 (1.64, 6.40)	3.09 (2.08, 4.60)	3.38 (2.13, 5.37)
Transgender/Other Gender Identity, Questioning	2.28 (1.32, 3.91)	3.51 (1.92, 6.46)	1.72 (1.49, 2.00)	1.38 (0.95, 2.02)	0.71 (0.47, 1.09)	3.51 (1.34, 9.16)
Depression						
Cisgender Male, Straight	Ref.	Ref.	Ref.	Ref.	Ref.	Ref.
Cisgender Male, Gay	1.84 (1.81, 1.87)*	1.63 (1.55, 1.71)	1.90 (1.83, 1.96)*	1.77 (1.71, 1.83)*	1.88 (1.77, 2.00)	1.68 (1.58, 1.79)
Cisgender Male, Bisexual	2.49 (2.31, 2.67)*	2.76 (2.39, 3.18)	2.49 (2.24, 2.77)	2.07 (1.96, 2.19)*	1.24 (1.10, 1.40)	3.03 (1.26, 4.08)
Cisgender male, Other sexual minority	1.82 (1.75, 1.88)*	1.43 (1.21, 1.69)	2.14 (2.04, 2.77)*	2.00 (1.86, 2.14)	1.45 (1.42, 1.48)*	1.41 (1.03, 1.92)
Cisgender Male, Questioning	1.25 (0.97, 1.60)	1.16 (0.78, 1.74)	1.78 (1.68, 1.88)	0.86 (0.50, 1.50)	0.86 (0.73, 1.01)	0.92 (0.42, 1.99)
Cisgender Female, Straight	1.24 (1.21, 1.28)	1.24 (1.20, 1.29)	1.25 (1.22, 1.27)*	1.21 (1.19, 1.24)	1.29 (1.26, 1.31)*	1.21 (1.17, 1.26)
Cisgender Female, Lesbian	1.89 (1.76, 2.02)	1.85 (1.55, 2.20)	1.93, 1.88, 1.99)*	1.71 (1.49, 1.95)	2.67 (2.41, 2.96)	1.28 (1.17, 1.41)
Cisgender Female, Bisexual	2.94 (2.80, 2.09)*	2.93 (2.50, 3.43)	2.86 (2.68, 3.05)*	2.53 (2.38, 2.68)*	3.65 (3.08, 4.31)	3.00 (2.72, 3.30)*
Cisgender Female, Other sexual minority	2.02 (1.94, 2.10)*	1.21 (0.98. 1.49)	2.45 (2.25, 2.67)	2.34 (2.10, 2.61)	1.76 (1.75, 1.77)*	3.90 (2.80, 5.43)
Cisgender Female, Questioning	1.57 (1.32, 1.87)	1.20 (0.95, 1.51)	2.09 (1.80, 2.44)	2.28 (1.44, 3.61)	0.96 (0.89, 1.04)	1.42 (1.37, 1.48)
Transgender/Other Gender Identity, Straight	1.08 (0.98, 1.18)	1.14 (1.10, 1.18)	1.02 (0.94, 1.11)	1.10 (1.10, 1.11)*	1.37 (0.95, 1.98)	1.77 (1.12, 2.80)
Transgender/Other Gender Identity, Gay or Lesbian	3.37 (1.18, 3.57)*	1.94 (1.69, 2.24)	4.08 (2.91, 5.74)	6.02 (4.32, 8.38)	4.85 (1.71, 13.82)	1.68 (1.06, 2.68)
Transgender/Other Gender Identity, Bisexual	6.22 (5.06, 7.64)	6.53 (5.98, 7.14)*	6.06 (5.01, 7.32)	2.78 (2.25, 3.44)	8.89 (7.66, 10.30)*	7.02 (3.67, 13.41)
Transgender/Other Gender Identity, Other Sexual Minority	3.21 (2.94, 3.49)*	2.62 (1.94, 3.52)	3.69 (3.38, 4.02)*	3.05 (2.49, 3.81)	3.60 (3.31, 3.92)*	1.76 (0.85, 3.63)
Transgender/Other Gender Identity, Questioning	2.16 (1.41, 3.31)	3.49 (1.58, 7.70)	1.71 (1.52, 1.92)	1.23 (0.94, 1.62)	0.79 (0.50, 1.26)	2.72 (1.23, 6.03)
b. Cisgender female, straight as reference.						
Anxiety						
Sexual Orientation, Gender Identity, and Race/ethnicity intersection	Overall OR (95% CI)	Hispanic OR (95% CI)	Non-Hispanic White OR (95% CI)	Non-Hispanic Black OR (95% CI)	Non-Hispanic Asian OR (95% CI)	Any other race or race combination OR (95% CI)

Fully Adjusted Model^a^						
Cisgender Female, Straight	Ref.	Ref.	Ref.	Ref.	Ref.	Ref.
Cisgender Male, Straight	0.59 (0.57, 0.60)*	0.62 (0.60, 0.63)*	0.57 (0.56, 0.58)*	0.63 (0.61, 0.64)*	0.59 (0.56, 0.61)*	0.57 (0.56, 0.59)*
Cisgender Male, Gay	1.14 (1.12, 1.16)	1.16 (1.05, 1.28)	1.11 (1.05, 1.17)	1.17 (1.14, 1.19)	1.08 (0.97, 1.21)	1.22 (1.18, 1.27)
Cisgender Male, Bisexual	1.38 (1.20, 1.59)	1.93 (1.16, 3.21)	1.30 (1.23, 1.37)	1.10 (0.91, 1.33)	0.86 (0.77, 0.96)	1.17 (0.82, 1.66)
Cisgender male, Other sexual minority	1.08 (1.04, 1.13)	0.92, (0.85, 0.99)	1.23 (1.12, 1.34)	1.34 (0.78, 2.31)	0.87 (0.77, 0.98)	0.74 (0.44, 1.26)
Cisgender Male, Questioning	0.78 (0.59, 1.03)	1.61 (1.12, 2.33)	1.03 (1.03, 1.34)*	0.69 (0.67, 0.70)*	0.56, 0.43, 0.73)	0.51 (0.26, 1.00)
Cisgender Female, Lesbian	1.50 (1.41, 1.59)	2.53 (2.05, 3.12)	1.50 (1.49, 1.51)*	1.24 (1.17, 1.31)	2.39 (2.17, 2.63)	1.06 (0.91, 1.23)
Cisgender Female, Bisexual	2.65 (2.60, 2.70)*	0.86 (0.66, 1.12)	2.60 (2.40, 2.83)*	2.37 (2.22, 2.52)*	2.50 (2.25, 2.79)	2.33 (2.18, 2.49)*
Cisgender Female, Other sexual minority	1.64 (1.35, 2.00)	0.69 (0.57, 0.83)	2.37 (2.34, 2.40)*	2.80 (2.09, 3.75)	1.20, 1.09, 1.31)	2.29 (2.27, 2.30)*
Cisgender Female, Questioning	1.01 (0.82, 1.24)	0.94 (0.72, 1.23)	1.58 (1.25, 2.00)	1.26 (0.80, 1.99)	0.72 (0.61, 0.84)	0.85 (0.80, 0.91)
Transgender/Other Gender Identity, Straight	0.75 (0.68, 0.84)	0.97 (0.62, 1.53)	0.68 (0.66, 0.70)*	0.98 (0.94, 1.01)	0.80 (0.56, 1.14)	0.75 (0.62, 0.92)
Transgender/Other Gender Identity, Gay or Lesbian	2.52 (2.15, 2.97)*	4.22 (3.50, 5.07)	3.55 (2.57, 4.90)	4.85 (3.71, 6.34)	10.94 (3.21, 37.30)	1.82 (1.38, 2.39)
Transgender/Other Gender Identity, Bisexual	4.16 (3.67, 4.70)	1.72 (1.41, 2.09)	3.98 (3.49, 4.54)	2.90 (2.83, 2.96)	2.00 (1.81, 2.22)	5.35 (1.98, 14.54)
Transgender/Other Gender Identity, Other Sexual Minority	1.82 (1.19, 2.78)	2.17 (1.21, 3.88)	1.62 (0.82, 3.21)	2.03 (1.00, 4.12)	1.81 (1.17, 2.80)	1.94 (1.18, 3.19)
Transgender/Other Gender Identity, Questioning	1.33 (0.79, 2.23)	1.16 (1.14, 1.17)	0.98 (0.86, 1.11)	0.90 (0.89, 0.90)	0.42 (0.26, 0.66)	2.01 (0.80, 5.09)
Depression						
Cisgender Female, Straight	Ref.	Ref.	Ref.	Ref.	Ref.	Ref.
Cisgender Male, Straight	0.81 (0.78, 0.83)	0.81 (0.78, 0.84)	0.80 (0.79, 0.82)*	0.83 (0.81, 0.84)	0.78 (0.76, 0.79)	0.82 (0.79, 0.86)*
Cisgender Male, Gay	1.48 (1.47, 1.50)*	1.31 (1.20, 1.43)	1.52 (1.49, 1.55)*	1.46 (1.44, 1.48)*	1.46 (1.40, 1.53)	1.39 (1.25, 1.54)
Cisgender Male, Bisexual	2.00 (1.9, 2.1)*	2.23 (1.86, 2.66)	2.00 (1.83, 2.18)	1.71 (1.65, 1.77)	0.96 (0.87, 1.07)	2.50 (1.93, 3.23)
Cisgender male, Other sexual minority	1.46 (1.45, 1.47)*	1.15 (1.01, 1.32)	1.72, (1.67, 1.77)*	1.65 (1.57, 1.73)	1.13 (1.09, 1.17)	1.16 (0.88, 1.52)
Cisgender Male, Questioning	1.0 (0.76, 1.33)	0.94, (0.60, 1.46)	1.43 (1.37, 1.48)	0.71 (0.40, 1.27)	0.67 (0.56, 0.80)	0.76 (0.34, 1.70)
Cisgender Female, Lesbian	1.52 (1.46, 1.59)	1.49 (1.30, 1.71)	1.55 (1.53, 1.56)*	1.41 (1.26, 1.57)	2.07 (1.90, 2.26)	1.06 (1.00, 1.11)
Cisgender Female, Bisexual	2.37 (2.32, 2.42)*	2.36 (1.95, 2.87)	2.29 (2.19, 2.40)*	2.09 (2.01, 2.17)*	2.84 (2.44, 3.29)	2.47 (2.33, 2.61)*
Cisgender Female, Other sexual minority	1.62 (1.52, 1.74)	0.98 (0.82, 1.16)	1.97 (1.77, 2.19)	1.93 (1.77, 2.11)	1.37 (1.34, 1.40)	3.21 (2.40, 4.30)
Cisgender Female, Questioning	1.27 (1.04, 1.55)	0.97 (0.74, 1.26)	1.68 (1.41, 1.99)	1.88 (1.21, 2.92)	0.75 (0.70, 0.80)	1.17 (1.08, 1.26)
Transgender/Other Gender Identity, Straight	0.87 (0.81, 0.93)	0.92 (0.86, 0.98)	0.82 (0.76, 0.88)	0.91 (0.89, 0.93)	1.07 (0.75, 1.51)	1.46 (0.96, 2.22)
Transgender/Other Gender Identity, Gay or Lesbian	2.71 (2.63, 2.79)*	1.57 (1.31, 1.87)	3.27 (2.38, 4.51)	4.97 (3.65, 6.77)	3.77 (1.35, 10.56)	1.38 (0.84, 2.29)*
Transgender/Other Gender Identity, Bisexual	5.00 (4.19, 5.98)	5.27 (5.00, 5.55)*	4.86 (4.10, 5.76)	2.29 (1.82, 2.90)*	6.91 (5.86, 8.15)	5.78 (3.15, 10.62)
Transgender/Other Gender Identity, Other Sexual Minority	2.58 (2.43, 2.73)*	2.11 (1.62, 2.74)	2.96 (2.66, 3.29)	2.52 (1.98, 3.22)	2.80 (2.53, 3.10)	1.45 (0.73, 2.88)
Transgender/Other Gender Identity, Questioning	1.74 (1.17, 2.59)	2.81 (1.32, 6.00)	1.37 (1.20, 1.57)	1.02 (0.76, 1.37)	0.62, 0.38, 0.99)	2.24 (1.05, 4.78)

a Adjusted for age, income, education level, recent job loss, and region.

* 0R Statistically significant after Bonferroni correction.
